# Study protocol of the PIMPI-project, a cohort study on acceptance, tolerability and immunogenicity of second trimester maternal pertussis immunization in relation to term and preterm infants

**DOI:** 10.1186/s12879-021-06559-w

**Published:** 2021-09-03

**Authors:** Maarten M. Immink, Mireille N. Bekker, Hester E. de Melker, Nynke Y. Rots, Elisabeth A. M. Sanders, Nicoline A. T. van der Maas

**Affiliations:** 1 Centre for Infectious Disease Control, National Institute for Public Health and the Environment (RIVM), Antonie van Leeuwenhoeklaan 9, 3720 MA Bilthoven, The Netherlands; 2 Department of Obstetrics, Wilhelmina Children’s Hospital, University Medical Center Utrecht, and Utrecht University, Utrecht, The Netherlands; 3 Department of Pediatrics, Wilhelmina Children’s Hospital, University Medical Center Utrecht, and Utrecht University, Utrecht, The Netherlands

**Keywords:** Pertussis, Maternal immunization, Vaccination, Prematurity, Acceptance, Tolerability, Immunogenicity

## Abstract

**Background:**

Maternal immunization confers passive immunity to the fetus by transplacental antibody transfer. Infants may be better protected against pertussis if the mother received a diphtheriae, tetanus and acellular pertussis (Tdap) vaccination in the second trimester of pregnancy compared to the third trimester. This study evaluates IgG antibody concentrations in term and preterm infants at birth and 2 months after birth after maternal Tdap-vaccination between 20^0^ and 24^0^ w of gestation vs third trimester Tdap-vaccination. Further aims are assessing the determinants that underlie acceptance of second trimester maternal Tdap-vaccination as well as the tolerability of vaccination.

**Methods:**

This prospective cohort study consists of two parts. In the acceptance part, pregnant women complete a questionnaire on determinants that underlie acceptance of a second trimester Tdap-vaccination, which is offered subsequently between 20^0^ and 24^0^ w of gestation. Vaccinated women complete an additional questionnaire on vaccination tolerability. Vaccinated women may also participate in the immunogenicity part, in which blood is drawn from mother at delivery and from infant at birth and 2 months after birth. Women are also eligible for the immunogenicity part if they received a Tdap-vaccination between 20^0^ and 24^0^ w of gestation via the national immunization program and get hospitalized for an imminent preterm delivery. Blood sampling continues until 60 term and 60 preterm mother-infant-pairs have been included. Pertussis-specific IgG antibody concentrations are determined in serum using a fluorescent bead-based multiplex immunoassay. For term infants, non-inferiority in IgG antibody concentrations against pertussis toxin (anti-PT) will be assessed referred to a historical control group in which mothers were Tdap-vaccinated between 30^0^ and 32^0^ w of gestation. For preterm infants, non-inferiority of anti-PT IgG concentrations is referred to as 85% of infants having ≥ 20 international units/mL at 2 months after birth.

**Discussion:**

This study investigates acceptance, tolerability and immunogenicity regarding maternal Tdap-immunization between 20^0^ and 24^0^ w of gestation. Its results provide insight into the effects of second trimester Tdap-vaccination on IgG antibody concentrations in term and preterm infants before primary infant vaccinations. Results on acceptance and tolerability guide antenatal care providers in communication with pregnant women and maintain the safety of second trimester Tdap-vaccination.

*Trial registration*: EU Clinical Trials Register, 2018-002976-41, retrospectively registered 24 July 2019, https://www.clinicaltrialsregister.eu/ctr-search/search?query=2018-002976-41.

**Supplementary Information:**

The online version contains supplementary material available at 10.1186/s12879-021-06559-w.

## Background

**Fig. 1:**
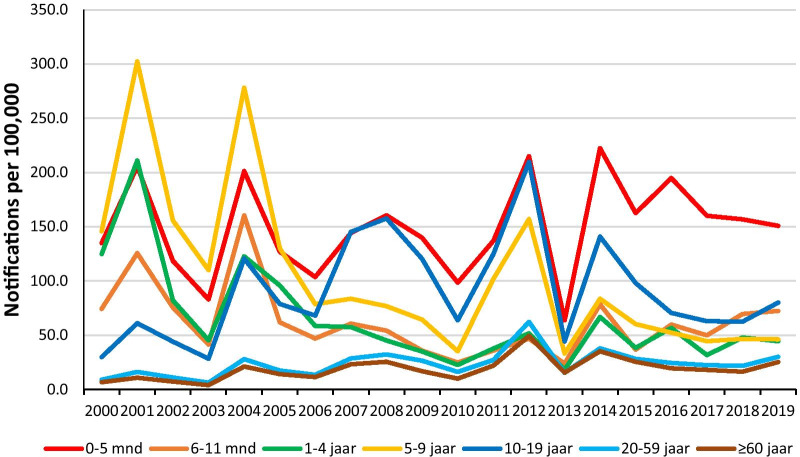
Number of pertussis notifications per 100,000 per age category in 2005–2019 [31]

Pertussis is a respiratory infectious disease caused mainly by *Bordetella pertussis*. Especially young infants are at increased risk of severe complications, hospitalization and sometimes even death [1]. Infant vaccinations against pertussis started around 1950 with steady high coverage, leading to lower incidences [2, 3]. However, in the nineties of the previous century, pertussis re-emerged in many countries, including the Netherlands [4]. Incidences in all ages increased with epidemic peaks every 3–4 years (Fig. 1) [5].

The Netherlands implemented several changes in the national immunization program in response to the increase. In 1999, the first pertussis containing vaccination was scheduled at 2 months (m) instead of 3 m. Late in 2001, an acellular pertussis component was added to the preschool diphtheria, tetanus and poliomyelitis booster dose at 4 years (y) of age and from 2005 onwards, the primary infant pertussis vaccinations also contained an acellular pertussis component instead of whole cell pertussis. However, surveillance data show that the incidence in young infants did not decrease following these changes (Fig. 1) [5]. In fact, an increase within this vulnerable age group was observed during every epidemic peak [2]. Adolescents and adults are likely a source of transmission to young infants [6].

In 2011, increased incidence rates of reported pertussis cases were followed by a large outbreak in 2012 in the Netherlands and surrounding countries, including England [7]. In response to an increasing number of pertussis related deaths, the English government decided to offer a maternal pertussis vaccination by means of a tetanus, diphtheria, acellular pertussis and poliomyelitis (Tdap-IPV) vaccine to all pregnant women during the third trimester. Maternal vaccination induces protection of young, not yet (fully) vaccinated infants. It confers passive immunity to the fetus by transplacental antibody transfer, which starts around 17 weeks (w) of gestational age (GA) and peaks in the third trimester [8]. After birth, maternal antibodies wane rapidly over time [9].

The uptake in England ranged around 70% and observational data showed a high vaccine effectiveness without any important safety concerns [9, 10]. To date, over 25 countries recommend maternal pertussis immunization with reassuring effectiveness and safety data. In December 2015, the Health Council of the Netherlands advised to offer a pertussis vaccination to all pregnant women in their third trimester [11]. In July 2018, the Ministry of Health, Welfare and Sports, decided to follow the advice of a maternal pertussis vaccination program. First vaccinations were offered mid December 2019.

Most countries offer third trimester Tdap-vaccination because of its benefits for newborns in general. However, preterm infants are less protected by third trimester Tdap-vaccination due to too short time intervals between vaccination and delivery. This was demonstrated in data from England, showing that preterm infants were overrepresented in pertussis hospitalizations and with an increase from 9.8 to 12.1% in the share of preterm infants after the introduction of third trimester Tdap-vaccination [12]. A similar overrepresentation of preterm infants among pertussis hospitalizations is shown in Norway (10.0% vs 5.2%) [13] and in the Netherlands (11.8% vs 7.8%) [14]. To offer women more opportunities for vaccination, England widened the interval for maternal Tdap-vaccination to the second trimester [15], resulting in an increase of the vaccination coverage of about 15% [16]. The overrepresentation of pertussis in preterm infants in England reduced strongly since widening this interval [17]. Switzerland also recommends vaccination from second trimester onwards and showed that infants of second trimester vaccinated mothers had higher antibody levels at birth than infants of third trimester vaccinated mothers [18]. Importantly, they also showed that preterm infants of second trimester vaccinated mothers had higher pertussis antibody levels at birth than preterm infants of third trimester vaccinated mothers [19]. By contrast, Winter et al. demonstrated that third trimester Tdap-vaccination was more effective in preventing clinical pertussis than vaccination earlier during pregnancy (85% vs 64%) [20]. However, both studies used different endpoints, i.e. Eberhardt et al. used immunogenicity as outcome measure, while Winter et al. used effectiveness as outcome measure. In the Netherlands, Tdap-vaccines are offered to pregnant women from 22 w GA onwards and administered via youth public healthcare services. Studies show that women in their second trimester are less willing to accept the vaccination compared to women later throughout gestation [21, 22]. However, these studies were performed without current knowledge that preterm infants are worse off than term infants following third trimester pertussis vaccination.

We set up this study of pregnant women and their infants to fill the knowledge gap on the effects of second trimester maternal Tdap-vaccination for the prevention of pertussis in term and preterm infants. In a prospective study that is divided into two study parts, i.e. acceptance and immunogenicity, we aim to assess the determinants that underlie acceptance of Tdap-vaccination in the second trimester of pregnancy and how second trimester Tdap-vaccination induces maternal antibody levels in term and preterm infants before primary vaccinations. Furthermore, we aim to assess tolerability of a maternal Tdap-vaccination.

## Methods/design

### Study design and objectives

This study is conducted as a prospective cohort study of pregnant women, with follow-up of their infants up to 2 m of age. It is divided into two parts. In the acceptance part, determinants that underlie acceptance of second trimester Tdap-vaccination are assessed using a questionnaire, as followed up by the tolerability after vaccination. In the immunogenicity part, pertussis-specific IgG antibody concentrations are determined in term and preterm infants at birth and 2 m after birth if the mother accepted the vaccination between 20^0^ and 24^0^ w GA.

#### Primary objectives

##### Immunogenicity part

- To evaluate non-inferiority of anti-Pertussis Toxin (PT) IgG antibodies in term infants at 2 m of age born of mothers who received a Tdap-vaccination between 20^0^ and 24^0^ w GA, compared to a reference anti-PT IgG at 2 m of age in a historical control group of term infants born of mothers who were vaccinated between 30^0^ and 32^0^ w GA in the period January 2014 to February 2016.
- To evaluate non-inferiority of anti-PT IgG in preterm infants at 2 m of age born of mothers who received a Tdap-vaccination between 20^0^ and 24^0^ w GA, as referred to at least 85% of preterm infants having anti-PT IgG concentrations ≥ 20 international units (IU)/mL as used in many immunogenicity studies.

#### Secondary objectives

##### Acceptance part

- To assess pregnant women’s social cognitive determinants and underlying beliefs on maternal Tdap-vaccination between 20^0^ and 24^0^ w GA and distinguish results among women who are pregnant for the first time, women who were pregnant before, and in both groups, women with and without a known increased risk of preterm delivery.
- To assess the correlation between social cognitive determinants and underlying beliefs and actual behavior, i.e. acceptance of a maternal Tdap-vaccination between 20^0^ and 24^0^ w GA.
- To compare social cognitive determinants and underlying beliefs of maternal Tdap-vaccination between 20^0^ and 24^0^ w GA with those of third trimester maternal vaccination.

##### Tolerability (extension of the acceptance part)

- To assess tolerability of the maternal Tdap-vaccination, administered to pregnant women between 20^0^ and 24^0^ w GA.
- To assess possible adverse pregnancy outcomes of maternal Tdap-vaccination between 20^0^ and 24^0^ w GA.

##### Immunogenicity part

- To compare pertussis specific IgG concentrations at 2 m of age (i.e. before the primary infant vaccinations) between term and preterm infants.
- To compare the decay in pertussis-specific maternal IgG concentrations in the first 2 m after birth between term and preterm infants.
- To compare pertussis-specific IgG concentrations at delivery between mothers who delivered term and preterm.
- To compare pertussis-specific IgG concentrations in mothers who received second trimester Tdap-vaccination and a group that received third trimester vaccination.
- To determine levels of pertussis-specific IgG transferred from the mother to the neonate relative to the interval from vaccination to delivery, if possible depending on the variation in interval.

### Study population and setting

The study population consists of pregnant women who receive primary, secondary or tertiary antenatal care. Participants are included by their antenatal care provider and followed-up prospectively in both the acceptance and immunogenicity study parts. By including women in secondary and tertiary care, we aim to oversample women with an increased risk for preterm delivery, e.g. women with multiple pregnancy, history of preterm delivery, cervical conization in the medical history and uterus anomaly.

In order to be eligible to participate in this study, women must be 18 y or older, pregnant, and in relation to the immunogenicity part of the study, both parents (or mother and legal guardian) must be willing to adhere to the protocol and perform all planned visits and sample collections for themselves and their newborn child. Women who meet any of the following criteria are excluded from participation in the immunogenicity part of this study: history of having received a pertussis containing vaccination in the past 2 years; known or suspected serious underlying condition that can interfere with the results of the study such as but not limited to cancer, autoimmune disease, immunodeficiency, seizure disorder or significant psychiatric illness; receipt of any high-dose (≥ 20 mg of prednisone daily or equivalent) daily corticosteroids within 2 weeks of study entry (inhaled or other local steroids are acceptable) with exception of corticosteroids to enhance maturation of fetal lungs in case of imminent early delivery; receipt of other immune modulating medication, for instance biologicals; receipt of blood products or immunoglobulins, within 3 months of study entry (rhesus negative women who receive anti-rhesus (D) immunoglobulin will not be excluded from the study); presence of bleeding disorder; having experienced a previous severe adverse reaction to any vaccine; receipt of any vaccine(s) within 2 weeks of study vaccine (except influenza vaccine which may be given concomitantly); all mothers who give birth before 24^0^ w GA.

### Recruitment and follow-up

In the acceptance part, women complete an online questionnaire on the determinants of acceptance of second trimester Tdap-vaccination. The vaccine is offered between 20^0^ and 24^0^ w GA and administered by their antenatal care provider if accepted. Vaccinated women complete a second questionnaire on the tolerability of vaccination. They are also eligible for participation in the immunogenicity study part. In this part, a blood sample from the mother and infant (cord blood) is drawn at delivery and a second sample from the infant at 2 m of age, i.e. before the first infant vaccination. Study samples are stored at the laboratory of the National Institute for Public Health and the Environment. Recruitment continues until blood samples are drawn from 60 term and 60 preterm mother-infant-pairs.

**Fig. 2:**
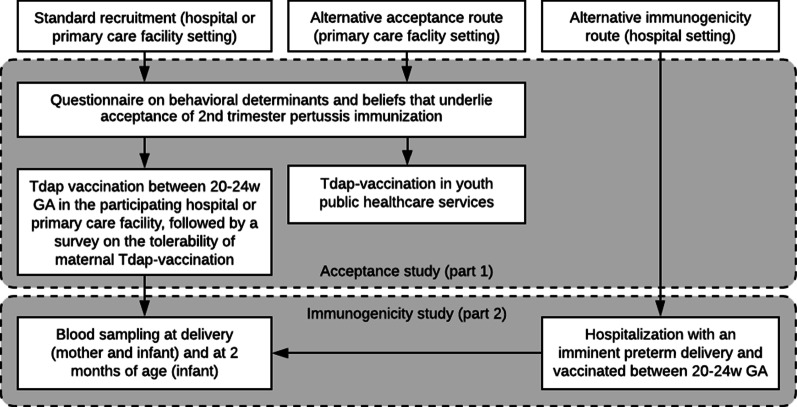
Participant recruitment and alternative recruitment routes in antenatal care in primary care facilities or hospitals

During the recruitment phase, two alternative recruitment routes were added to increase inclusion speed for both study parts (Fig. 2). The alternative acceptance route focuses on recruitment of women for the acceptance part via midwives in primary care facilities. The alternative immunogenicity route focuses on faster recruitment of preterm infants via secondary and tertiary antenatal care.

#### Alternative acceptance route

After completing the questionnaire on the determinants that underlie acceptance of second trimester Tdap-vaccination, midwifes inform pregnant women about the possibility of getting a Tdap-vaccination via youth public healthcare services. Vaccination status is requested retrospectively via the national immunization registry. The vaccine is not administered by the antenatal care provider in this route.

#### Alternative immunogenicity route

Antenatal care providers in hospitals ask consent for sampling cord blood and finger-stick-blood if a women gets hospitalized for an imminent preterm delivery and proves that she received a Tdap-vaccination between 20^0^ and 24^0^ w GA via the national immunization program. In case of an unclear answer, vaccination status is requested retrospectively via the national immunization registry. Further study procedures for blood sampling are identical to those in in the immunogenicity part.

### Questionnaires

#### Acceptance

The online questionnaire focuses on behavioral determinants and beliefs that underlie acceptance of a second trimester Tdap-vaccination. It consists of about sixty questions and statements assessing demographics, social cognitive determinants, underlying beliefs, past experiences, women’s information desires and considerations regarding information provision and implementation of maternal Tdap-vaccination. A Dutch version of the questionnaire is provided in Additional file 1: Appendix 1.

#### Tolerability

This questionnaire contains questions about the onset of local reactions and solicited systemic adverse events (AE) within 1 week post-vaccination. Local reactions include swelling, redness, and pain at the injection site. Systemic AEs include fever, headache, tiredness, nausea, vomiting, diarrhea, dizziness, loss of appetite, stiffness of muscles and joints, itch, abnormal sweating, skin rash, swollen lymph nodes, sore throat, upper airway infection, coughing, fainting, and influenza-like illness. The questionnaire also includes questions about solicited systemic events at baseline, i.e. in the week pre-vaccination. Time interval and duration of symptoms are collected, as well as the use of analgesics, medical intervention, and absence from work and/or other activities. A Dutch version of the survey is provided in Additional file 2: Appendix 2.

### Blood sample collection infant and mother (heel or finger stick)

For IgG testing using the X-map Luminex technology, a maximum of 2 mL infant cord blood is used. Furthermore, a maximum of 300 µl blood samples of the mother at birth and of the infants at 2 m of age is collected by heel or finger stick. Samples are tested for IgG antibody concentrations against pertussis antigens, diphtheria and tetanus. Antibody concentrations will be assessed in serum using a fluorescent bead-based multiplex immunoassay [23].

### Defining prematurity

We define preterm infants as infants born before 35^0^ w GA, although normally the cut-off for prematurity is set before 37^0^ w GA. We assume that for preterm infants born between 35^0^ and 36^0^ w GA second or third trimester maternal Tdap-vaccination will generally allow enough time for sufficient transfer of antibodies. We will use the same definition for term infants as used in the historical control group, i.e. ≥ 37^0^ w GA.

### Investigational product

Boostrix is a suspension for injection in a prefilled syringe containing diphtheria, tetanus and acellular pertussis vaccine (adsorbed, reduced antigen content). The Boostrix vaccine will be given as a single 0.5 mL intramuscular injection, in the deltoid muscle of the upper arm between 20^0^ and 24^0^ w GA. For subjects who are ill or have a moderate or high fever (rectal temperature of > 38.0 °C), vaccination will be postponed until the symptoms of illness and the fever have disappeared. Pregnant women in the historical cohort [24] received the same vaccine investigational product.

### Sample size calculation for immunogenicity part

To reach non-inferiority in term infants, the lower limit of the 95% confidence interval (CI) of the geometric mean concentration (GMC) after second trimester vaccination divided by the GMC after third trimester vaccination must be ≥ 0.5, with a one-sided 2.5% significance level and 80% power. As the GMC of anti-PT IgG in the historical control group (n = 58) was 26.1 IU/mL (95% CI 19.5–35.0), we need to include 53 term infants in the study. Taking into account 10% drop out or failed blood sampling, 58 term infants suffice.

Furthermore, non-inferiority in preterm infants is defined as 85% of infants with an anti-PT IgG above the 20 IU/mL at 2 m of age. This cut-off is used in many immunogenicity studies. With 10% precision, we need to include 49 blood samples from preterm mother-infant-pairs. Taking into account 10% drop-out or failed blood sampling, 54 preterm infants suffice. Due to the probability of multiple pregnancies in the preterm infant group and the likeliness of correlation between twins and triplets, preterm mother-infant-pairs are included and counted as one after multiple birth.

### Statistical analyses

#### Acceptance part

Items targeting social cognitive determinants and beliefs are measured on 7-point Likert scales. Items with the same underlying theoretical construct will be averaged into one single construct in case internal consistency is sufficient (Cronbach’s alpha α > 0.60 or Pearson correlation coefficient r > 0.50). Spearman’s correlation test will be used to explore univariate associations for attitude with social cognitive determinants, underlying beliefs and possible barriers and facilitators. We will control for the false discovery rate in multiple testing according to the Benjamini–Hochberg procedure. Next, variables with the largest predictive value for women’s attitude towards pertussis vaccination during pregnancy will be determined by random forest analysis. We will compare results with a similar study which assessed the determinants of acceptance of third trimester maternal vaccination, to distinguish determinants on the acceptance of maternal Tdap-vaccination in second versus third trimester of pregnancy [25].

#### Tolerability (extension of the acceptance part)

The percentage and 95% CI of pregnant women experiencing adverse events (AE) within 1 week after Tdap-vaccination are described by type and severity of the AE. Using binary generalized mixed models (GLMM), the association between the occurrence of symptoms in the week before and the week after vaccination will be analyzed. Proportions of absence from work and/or other activities, and medical intervention within 7 days after vaccination will be calculated together with 95% CI as well as the association of these items before and after vaccination using GLMM.

#### Immunogenicity part

For term and preterm infants, GMCs and 95% CIs will be calculated for IgG antibodies against three pertussis antigens in the vaccine (PT, filamentous hemagglutinin (FHA), pertactin (Prn)), tetanus and diphtheria in mothers and infants at delivery, and for infants at 2 m of age, before the start of infant vaccination. Differences in GMCs between the two groups will be analyzed with a t-test. All reported p-values are 2-sided, p-values ≥ 0.05 are considered significant.

The decay in IgG antibody concentrations against PT, Prn, FHA, tetanus and diphtheria from birth until 2 m of age will be analyzed with a paired t-test that compares GMCs at birth and at 2 m of age, for term and preterm infants separately. The ratio between maternal GMCs and infant GMCs at birth will be calculated for term and preterm infants separately and stratified for time interval between vaccination and delivery.

For term infants, we will compare the results with another maternal Tdap-vaccination trial in which pregnant women received a Tdap-vaccination between 30^0^ and 32^0^ w GA. Anti-PT IgG concentrations of the 58 term infants from the comparator trial were also measured at 2 m of age.

## Discussion

### General implications of results

This prospective cohort study will investigate the acceptance, tolerability and immunogenicity regarding maternal Tdap-vaccination between 20^0^ and 24^0^ w GA. Its results will provide valuable insights into anti-PT IgG antibody concentrations in term and preterm infants before primary infant vaccinations and how the vaccination schedule of these infants may be optimized in response to second trimester Tdap-vaccination. Determinants and beliefs that underlie pregnant women’s acceptance of second trimester Tdap-vaccination may guide antenatal care providers in the communication and recommendation of vaccinating early throughout gestation. Furthermore, assessing local reactions and solicited systemic adverse events following second trimester Tdap-vaccination will aid in communication about its safety profile.

Changes in the Dutch vaccination schedule may be considered if non-inferiority of anti-PT IgG antibodies in preterm infants at 2 m is addressed. Currently in the Netherlands, a Tdap-vaccination during pregnancy replaces the first infant vaccination for term infants who may then be vaccinated at 3, 5 and 11 m with a DTaP-IPV-Hib-HepB vaccine. Preterm infants are excluded from this reduced schedule and receive an extra vaccination at 2 m of age. GMCs of preterm infants at 2 m of age may inform us whether such a reduced schedule is also feasible for (late) preterm infants if they relate to GMCs at corresponding ages in term infants. For term infants, non-inferiority of anti-PT IgG antibody concentrations after second trimester Tdap-vaccination informs us that anti-PT IgG antibodies may still be higher at 2 m of age, compared to third trimester Tdap-vaccination. Since more than 93% of all annual births in the Netherlands are born after 37^0^ w GA [26], the results of this study may provide reassurance for pregnant women to obtain the maternal Tdap-vaccination at the earliest opportunity throughout gestation, i.e. at 22 w GA in the Netherlands.

### Strengths

This is the first time that anti-PT IgG concentrations are investigated in term and preterm infants up to 2 m of age in response to second trimester Tdap-vaccination. The prospective study design with measurements at birth and 2 m of age allows us to assess the velocity of antibody decay before primary infant vaccinations. Drawing blood samples from mother-infant-pairs at delivery provides additional insight in the rate of placental antibody transfer corrected for gestation. Combining these aspects in immunogenicity with the acceptance part into a single study design results in efficient follow-up of women participating in both study parts and also complete the questionnaire on tolerability.

To our knowledge, behavioral determinants and beliefs that underlie maternal Tdap-vaccination acceptance have not yet been assessed specific for administering the vaccine in the second trimester of pregnancy. The use of multiple recruitment routes allows us to assess acceptance in women in primary care facilities and hospitals, thus reaching both healthy pregnant women and women with an increased risk of preterm delivery. The results may ultimately be used for communication early in pregnancy, especially when the risk of preterm delivery is addressed.

### Limitations

Assessing antibodies in term mother-infant-pairs based on a historical comparator results in performing analyses at different timepoints, which might affect pertussis antibody responses. However, the differences between these cohorts are limited to the time interval of Tdap-vaccination throughout gestation in both studies. Remaining study procedures, e.g. recruitment, data management and the used investigational product, are similar and performed by the same research institute and laboratory.

Since a maternal Tdap-vaccine could be obtained free of charge via study participation, pregnant women with high intention of acceptance may introduce selection bias due to high willingness of participation. After December 2019 when the vaccination is offered within the National Immunization Program for which no money is charged, women may still be likely to participate in this study for obtaining the vaccine from their antenatal care provider, instead of making an appointment for vaccination at a youth public healthcare service. Altogether, the results of second trimester Tdap-acceptance among pregnant women may be estimated more optimistic than in real life.

The results of our study are limited to a follow-up time of 2 m after birth. Maternal antibodies are known to interfere with term infants’ immune responses after primary vaccination series, which is known as blunting [24, 27–29]. The likeliness of a reduced immune response after primary vaccinations is not assessed in infants born of mothers who received second trimester maternal Tdap-vaccination and remains implicated for future research.

## Supplementary Information

**Additional file 1: Appendix 1.** Questionnaire on determinants that underlie acceptance of early maternal pertussis immunization (in Dutch).

**Additional file 2: Appendix 2.** Questionnaire on local reactions and solicited systemic adverse events before and after vaccination (in Dutch).

## Data Availability

The anonymized datasets generated during the current study will be made available from the corresponding author on reasonable request.

## Abbreviations

AE: Adverse event
CI: Confidence interval
FHA: Filamentous hemagglutinin
GA: Gestational age
GMC: Geometric mean concentration
IU: International units
m: Months
Prn: Pertactin
PT: Pertussis toxin
Tdap: Tetanus, diphtheriae, acellular pertussis
w: Weeks
y: Years
