# Supplementary material for: Study protocol of the PIMPI-project, a cohort study on acceptance, tolerability and immunogenicity of second trimester maternal pertussis immunization in relation to term and preterm infants
Source: BMC Infect Dis. 2021 Sep 3;21:897. doi: 10.1186/s12879-021-06559-w (PMC8414744; doi:10.1186/s12879-021-06559-w)
Supplement: Supplementary file 1 — Additional file 1: Appendix 1. Questionnaire on determinants that underlie acceptance of early maternal pertussis immunization (in Dutch). [file 12879_2021_6559_MOESM1_ESM.docx]

**Appendix 1.** Questionnaire on determinants that underlie acceptance of early maternal pertussis immunization (in Dutch)

| **Vragen** | | **Antwoordmogelijkheden** |
| --- | --- | --- |
| **Persoonlijke informatie** | | |
| Op welke datum bent u uitgerekend? | | <datum> |
| Wat zijn de 4 cijfers van uw postcode? | | <getal vier cijfers> |
| Wat is uw leeftijd in jaren? | | <getal twee cijfers> |
| Wat is uw geboorteland? | | Nederland, Suriname; (voormalige) Nederlandse Antillen; Turkije; Marokko; anders |
| Wat is uw hoogst voltooide opleiding? | | geen opleiding (lager onderwijs niet afgemaakt); lager onderwijs (basisschool, speciaal basisonderwijs); lager of voorbereidend beroepsonderwijs (zoals LTS, VMBO-basis, kader of GL); middelbaar algemeen voortgezet onderwijs (zoals MAVO, MBO­-kort, VMBO­-TL); middelbaar beroepsonderwijs (MBO); hoger algemeen en voorbereidend wetenschappelijk onderwijs (zoals HAVO, VWO, atheneum, gymnasium); hoger beroepsonderwijs (HBO); wetenschappelijk onderwijs (universiteit) |
| Voor de hoeveelste keer bent u zwanger? | | 1e; 2e; 3e; 4e; 5e; 6e; 7e; 8e; 9e; 10e of vaker |
| Hoeveel eigen kinderen heeft u? | | 1; 2; 3; 4; 5; 6; 7; 8; 9; 10 of meer |
| Wat is de geboortedatum van uw (jongste) kind?^1^ | | <datum> |
| Doet uw (jongste) kind mee aan het Rijksvaccinatieprogramma?^1^ | | ja, volledig (alle vaccinaties gekregen die het voor zijn/haar leeftijd zou moeten hebben); ja, gedeeltelijk (niet alle vaccinaties gekregen die het voor zijn/haar leeftijd zou moeten hebben); nee; weet ik niet |
| Tijdens mijn zwangerschap sta ik onder controle bij: | | verloskundige; klinisch/tweedelijns verloskundige; arts-assistent van het ziekenhuis; gynaecoloog |
| De onderstaande overtuigingen zijn van toepassing op mij: | |  |
|  | Geloofsovertuiging | 1 (helemaal niet van toepassing); 2; 3; 4; 5; 6; 7 (heel erg van toepassing) |
|  | Homeopathie | 1 (helemaal niet van toepassing); 2; 3; 4; 5; 6; 7 (heel erg van toepassing) |
|  | Natuurgeneeswijzen | 1 (helemaal niet van toepassing); 2; 3; 4; 5; 6; 7 (heel erg van toepassing) |
|  | Antroposofie | 1 (helemaal niet van toepassing); 2; 3; 4; 5; 6; 7 (heel erg van toepassing) |
| **Kinkhoest en kinkhoestvaccinatie tijdens de zwangerschap** | | |
| Hoe ernstig zijn de gevolgen van kinkhoest voor baby’s volgens u? | | 1 (niet ernstig); 2; 3; 4; 5; 6; 7 (zeer ernstig) |
| Stel dat u zich niet tijdens de zwangerschap tegen kinkhoest laat vaccineren, hoe groot acht u dan de kans dat uw baby kinkhoest krijgt? | | 1 (zeer klein); 2; 3; 4; 5; 6; 7 (zeer groot) |
| Stel dat u zich tijdens de zwangerschap tegen kinkhoest laat vaccineren, hoe groot acht u dan de kans op negatieve gevolgen voor het verloop van uw zwangerschap? | | 1 (zeer klein); 2; 3; 4; 5; 6; 7 (zeer groot) |
| Stel dat u zich tijdens de zwangerschap tegen kinkhoest laat vaccineren, hoe groot acht u dan de kans dat uw baby later last krijgt van bijwerkingen? | | 1 (zeer klein); 2; 3; 4; 5; 6; 7 (zeer groot) |
| Stel dat u zich tijdens de zwangerschap tegen kinkhoest laat vaccineren, hoe groot acht u dan de kans dat u bijwerkingen krijgt? | | 1 (zeer klein); 2; 3; 4; 5; 6; 7 (zeer groot) |
| Hoe ernstig zijn de bijwerkingen van de kinkhoestvaccinatie tijdens de zwangerschap voor uzelf volgens u? | | 1 (niet ernstig); 2; 3; 4; 5; 6; 7 (zeer ernstig) |
| Hoe ernstig zijn de bijwerkingen van de kinkhoestvaccinatie tijdens de zwangerschap voor uw baby volgens u? | | 1 (niet ernstig); 2; 3; 4; 5; 6; 7 (zeer ernstig) |
| Ik vind dat het doormaken van kinkhoest bijdraagt aan een positieve mentale en lichamelijke ontwikkeling van mijn baby. | | 1 (helemaal mee oneens); 2; 3; 4; 5; 6; 7 (helemaal mee eens) |
| Ik vind dat het doormaken van kinkhoest positief is voor mijn baby. | | 1 (helemaal mee oneens); 2; 3; 4; 5; 6; 7 (helemaal mee eens) |
| Ik vind dat de kinkhoestvaccinatie tijdens de zwangerschap een goede manier is om negatieve gevolgen van kinkhoest bij baby’s te voorkomen. | | 1 (helemaal mee oneens); 2; 3; 4; 5; 6; 7 (helemaal mee eens) |
| Ik denk dat de kinkhoestvaccinatie tijdens de zwangerschap veilig is voor de zwangere vrouw. | | 1 (helemaal mee oneens); 2; 3; 4; 5; 6; 7 (helemaal mee eens) |
| Ik denk dat de kinkhoestvaccinatie tijdens de zwangerschap veilig is voor de baby. | | 1 (helemaal mee oneens); 2; 3; 4; 5; 6; 7 (helemaal mee eens) |
| Ik vind dat het kinkhoestvaccin nog onvoldoende op veiligheid is getest bij zwangere vrouwen. | | 1 (helemaal mee oneens); 2; 3; 4; 5; 6; 7 (helemaal mee eens) |
| Ik vind dat vaccinaties onvoldoende beschermen tegen de infectieziekten waartegen gevaccineerd wordt. | | 1 (helemaal mee oneens); 2; 3; 4; 5; 6; 7 (helemaal mee eens) |
| Ik denk dat er stoffen in vaccins zitten die schadelijk kunnen zijn voor de gezondheid van mijn baby. | | 1 (helemaal mee oneens); 2; 3; 4; 5; 6; 7 (helemaal mee eens) |
| Ik denk dat de kinkhoestvaccinatie aan zwangere vrouwen wordt aangeboden zodat de farmaceutische industrie hier geld aan kan verdienen. | | 1 (helemaal mee oneens); 2; 3; 4; 5; 6; 7 (helemaal mee eens) |
| Ik denk dat als de kinkhoestvaccinatie al in andere landen (bijv. Engeland en België) aan zwangere vrouwen wordt aangeboden, dat de vaccinatie dan veilig zal zijn. | | 1 (helemaal mee oneens); 2; 3; 4; 5; 6; 7 (helemaal mee eens) |
| Ik denk dat vaccineren tegen kinkhoest tijdens de zwangerschap zorgt voor minder kinkhoest bij baby’s | | 1 (helemaal mee oneens); 2; 3; 4; 5; 6; 7 (helemaal mee eens) |
| Ik denk dat door het vaccineren tegen kinkhoest tijdens de zwangerschap, baby’s beschermd zijn totdat ze zelf gevaccineerd kunnen worden. | | 1 (helemaal mee oneens); 2; 3; 4; 5; 6; 7 (helemaal mee eens) |
| Ik denk dat vaccineren tegen kinkhoest tijdens de zwangerschap zorgt voor meer complicaties tijdens mijn zwangerschap. | | 1 (helemaal mee oneens); 2; 3; 4; 5; 6; 7 (helemaal mee eens) |
| Ik denk dat vaccineren tegen kinkhoest tijdens de zwangerschap zorgt voor een minder goede weerstand van mijn baby. | | 1 (helemaal mee oneens); 2; 3; 4; 5; 6; 7 (helemaal mee eens) |
| Ik denk dat er een goed alternatief is voor kinkhoestvaccinatie tijdens de zwangerschap. | | 1 (helemaal mee oneens); 2; 3; 4; 5; 6; 7 (helemaal mee eens) |
| **Keuze wel of niet te laten vaccineren tijdens de zwangerschap** | | |
| Ik vind het advies van de verloskundige of gynaecoloog om mij te laten vaccineren tegen kinkhoest tijdens mijn zwangerschap | | 1 (helemaal niet belangrijk); 2; 3; 4; 5; 6; 7 (heel erg belangrijk) |
| Ik vind het advies van mijn partner om mij te laten vaccineren tegen kinkhoest tijdens mijn zwangerschap | | 1 (helemaal niet belangrijk); 2; 3; 4; 5; 6; 7 (heel erg belangrijk) |
| Ik denk dat de mensen die belangrijk voor mij zijn het zullen waarderen als ik mij tijdens de zwangerschap tegen kinkhoest laat vaccineren. | | 1 (helemaal mee oneens); 2; 3; 4; 5; 6; 7 (helemaal mee eens) |
| Ik denk dat mijn verloskundige of gynaecoloog vindt dat ik mij tijdens de zwangerschap tegen kinkhoest moet laten vaccineren. | | 1 (helemaal mee oneens); 2; 3; 4; 5; 6; 7 (helemaal mee eens) |
| Ik denk dat de meeste zwangere vrouwen zich tijdens de zwangerschap tegen kinkhoest laten vaccineren. | | 1 (helemaal mee oneens); 2; 3; 4; 5; 6; 7 (helemaal mee eens) |
| Ik vind dat het bij mijn verantwoordelijkheid als zwangere hoort om mij tijdens de zwangerschap tegen kinkhoest te laten vaccineren om mijn baby te beschermen. | | 1 (helemaal mee oneens); 2; 3; 4; 5; 6; 7 (helemaal mee eens) |
| Ik vind dat het bij mijn verantwoordelijkheid als zwangere hoort om de kinkhoestvaccinatie tijdens mijn zwangerschap te weigeren. | | 1 (helemaal mee oneens); 2; 3; 4; 5; 6; 7 (helemaal mee eens) |
| Ik vind dat het bij de verantwoordelijkheid van iedere zwangere hoort om zich tijdens de zwangerschap tegen kinkhoest te laten vaccineren om hun baby te beschermen. | | 1 (helemaal mee oneens); 2; 3; 4; 5; 6; 7 (helemaal mee eens) |
| Ik vind dat het bij de verantwoordelijkheid van iedere zwangere hoort om de kinkhoestvaccinatie tijdens hun zwangerschap te weigeren. | | 1 (helemaal mee oneens); 2; 3; 4; 5; 6; 7 (helemaal mee eens) |
| Stel dat u zich tijdens de zwangerschap niet tegen kinkhoest laat vaccineren en uw baby krijgt later kinkhoest. Hoeveel spijt zult u dan hebben van uw besluit zich niet te laten vaccineren? | | 1 (helemaal geen spijt); 2; 3; 4; 5; 6; 7 (heel erg veel spijt) |
| Stel dat u zich tijdens de zwangerschap wel tegen kinkhoest laat vaccineren en uw baby krijgt last van bijwerkingen. Hoeveel spijt zult u dan hebben van uw besluit zich te laten vaccineren? | | 1 (helemaal geen spijt); 2; 3; 4; 5; 6; 7 (heel erg veel spijt) |
| Mij laten vaccineren tegen kinkhoest tijdens de zwangerschap is iets waar ik lang over na moet denken. | | 1 (helemaal mee oneens); 2; 3; 4; 5; 6; 7 (helemaal mee eens) |
| Ik ben mij bewust van de voor- en nadelen van het vaccineren tegen kinkhoest tijdens mijn zwangerschap. | | 1 (helemaal mee oneens); 2; 3; 4; 5; 6; 7 (helemaal mee eens) |
| Ik heb getwijfeld over het wel of niet laten vaccineren tegen kinkhoest tijdens mijn zwangerschap. | | 1 (helemaal mee oneens); 2; 3; 4; 5; 6; 7 (helemaal mee eens) |
| **Keuze wel of niet te vaccineren tijdens de zwangerschap (vervolg)** | |  |
| Als ik eraan denk om mij tijdens mijn zwangerschap te laten vaccineren, dan vind ik dat: | | 1 (helemaal niet eng); 2; 3; 4; 5; 6; 7 (heel erg eng) |
|  | Wat vindt u eng als u aan vaccineren tijdens de zwangerschap denkt?^2^ | <open invulveld> |
| Als ik eraan denk dat mijn pasgeboren baby kinkhoest zou kunnen krijgen, dan vind ik dat: | | 1 (helemaal niet eng); 2; 3; 4; 5; 6; 7 (heel erg eng) |
|  | Wat vindt u eng als u eraan denkt dat uw baby kinkhoest zou kunnen krijgen?^3^ | <open invulveld> |
| Als er een alternatief is voor het vaccineren tegen kinkhoest tijdens de zwangerschap, dan zal ik daarvoor kiezen. | | 1 (helemaal mee oneens); 2; 3; 4; 5; 6; 7 (helemaal mee eens) |
| Ik vind dat ongeboren baby’s te kwetsbaar zijn om ze al tijdens de zwangerschap te belasten met een vaccin. | | 1 (helemaal mee oneens); 2; 3; 4; 5; 6; 7 (helemaal mee eens) |
| Ik vind dat vaccineren tijdens de zwangerschap tegenstrijdig is met zo min mogelijk medicijngebruik tijdens de zwangerschap. | | 1 (helemaal mee oneens); 2; 3; 4; 5; 6; 7 (helemaal mee eens) |
| Ik vind het vaccineren tegen kinkhoest tijdens mijn zwangerschap: | | 1 (heel erg slecht); 2; 3; 4; 5; 6; 7 (heel erg goed) |
| Ik vind het vaccineren tegen kinkhoest tijdens mijn zwangerschap: | | 1 (heel erg onbelangrijk); 2; 3; 4; 5; 6; 7 (heel erg belangrijk) |
| Ik vind het vaccineren tegen kinkhoest tijdens mijn zwangerschap: | | 1 (heel erg onnodig); 2; 3; 4; 5; 6; 7 (heel erg nodig) |
| Ik ben van plan mij tijdens mijn zwangerschap tegen kinkhoest te laten vaccineren. | | 1 (zeker niet); 2; 3; 4; 5; 6; 7 (zeker wel) |
| Ik verwacht dat ik mij tijdens mijn zwangerschap tegen kinkhoest zal laten vaccineren. | | 1 (zeker niet); 2; 3; 4; 5; 6; 7 (zeker wel) |
| Het is waarschijnlijk dat ik mij tijdens mijn zwangerschap tegen kinkhoest zal laten vaccineren. | | 1 (zeker niet); 2; 3; 4; 5; 6; 7 (zeker wel) |
| **Informatiebehoefte en praktische overwegingen** | | |
| Hoeveel vertrouwen heeft u in de informatie die u van uw verloskundige of gynaecoloog krijgt over de kinkhoestvaccinatie tijdens de zwangerschap? | | 1 (helemaal geen vertrouwen); 2; 3; 4; 5; 6; 7 (heel veel vertrouwen) |
| Hoeveel vertrouwen heeft u in informatie van het RIVM over de kinkhoestvaccinatie tijdens de zwangerschap? | | 1 (helemaal geen vertrouwen); 2; 3; 4; 5; 6; 7 (heel veel vertrouwen) |
| Hoeveel vertrouwen heeft u in het besluit van de overheid om een kinkhoestvaccinatie tijdens de zwangerschap aan te bieden? | | 1 (helemaal geen vertrouwen); 2; 3; 4; 5; 6; 7 (heel veel vertrouwen) |
| Ik vind het niet fijn dat de kinkhoestvaccinatie een combinatievaccin is, waardoor ik ook gevaccineerd word tegen andere infecties (difterie en tetanus). | | 1 (helemaal mee oneens); 2; 3; 4; 5; 6; 7 (helemaal mee eens) |
| Ik vind het fijn als mijn kind iets later kan beginnen met de vaccinaties en een prik minder nodig heeft, doordat ik me tijdens de zwangerschap laat vaccineren. | | 1 (helemaal mee oneens); 2; 3; 4; 5; 6; 7 (helemaal mee eens) |
| Ik voel mij door anderen onder druk gezet bij het maken van een keuze rondom het vaccineren tegen kinkhoest tijdens mijn zwangerschap. | | 1 (helemaal mee oneens); 2; 3; 4; 5; 6; 7 (helemaal mee eens) |
| Dit waren alle vragen. Als u nog opmerkingen heeft over de vragenlijst, dan kunt u dat hier aangeven. | | <open invulveld> |

^1^Vraag van toepassing als ‘Hoeveel eigen kinderen heeft u?’ is meer dan 1. ^2^Vraag van toepassing als ‘Als ik eraan denk om mij tijdens mijn zwangerschap te laten vaccineren, dan vind ik dat:’ is 4 of hoger. ^3^Vraag van toepassing als ‘Als ik eraan denk dat mijn pasgeboren baby kinkhoest zou kunnen krijgen, dan vind ik dat:’ is 4 of hoger.
