# Supplementary material for: Study protocol of the PIMPI-project, a cohort study on acceptance, tolerability and immunogenicity of second trimester maternal pertussis immunization in relation to term and preterm infants
Source: BMC Infect Dis. 2021 Sep 3;21:897. doi: 10.1186/s12879-021-06559-w (PMC8414744; doi:10.1186/s12879-021-06559-w)
Supplement: Supplementary file 2 — Additional file 2: Appendix 2. Questionnaire on local reactions and solicited systemic adverse events before and after vaccination (in Dutch). [file 12879_2021_6559_MOESM2_ESM.docx]

**Appendix 2.** Questionnaire on local reactions and solicited systemic adverse events before and after vaccination (in Dutch)

| **Vragen** | | **Antwoordmogelijkheden** |
| --- | --- | --- |
| **Persoonsgegevens** | | |
| Op welke datum kreeg u de kinkhoestvaccinatie? | | <datum> |
| Heeft u een chronische ziekte/aandoening? | | ja; nee |
|  | Welke? | <open invulveld> |
|  | Gebruikt u hiervoor medicijnen? | ja; nee |
|  | Welke medicijnen? | <open invulveld> |
| Heeft u een aandoening die invloed kan hebben op uw zwangerschap? | | ja; nee |
|  | Welke? | <open invulveld> |
| **Symptomen in de week vóór de vaccinatie**  Geef hieronder aan of u symptomen had in de week vóórafgaande aan de vaccinatie. Als u bij de symptomen 'ja' invult, beantwoord dan ook de vervolgvragen. | | |
| Had u last van koorts? | | ja; nee |
|  | Wat was de hoogst gemeten temperatuur? | 38 tot 39°C; 39 tot 40°C; 40 tot 41°C; hoger dan 41°C |
|  | Hoe is dit gemeten? | rectaal; oksel; oor; tast; anders; niet gemeten |
|  | Wanneer is dit begonnen? | <datum> |
|  | Hoeveel dagen heeft het geduurd? | 1; 2; 3; 4; 5; 6; 7; meer dan 7 |
|  | Toelichting | <open invulveld> |
| Had u last van hoofdpijn? | | ja; nee |
|  | Hoe ernstig was het? | iets; matig; veel |
|  | Wanneer is dit begonnen? | <datum> |
|  | Hoeveel dagen heeft het geduurd? | 1; 2; 3; 4; 5; 6; 7; meer dan 7 |
|  | Toelichting | <open invulveld> |
| Had u last van vermoeidheid? | | ja; nee |
|  | Hoe ernstig was het? | iets; matig; veel |
|  | Wanneer is dit begonnen? | <datum> |
|  | Hoeveel dagen heeft het geduurd? | 1; 2; 3; 4; 5; 6; 7; meer dan 7 |
|  | Toelichting | <open invulveld> |
| Had u last van misselijkheid? | | ja; nee |
|  | Hoe ernstig was het? | iets; matig; veel |
|  | Wanneer is dit begonnen? | <datum> |
|  | Hoeveel dagen heeft het geduurd? | 1; 2; 3; 4; 5; 6; 7; meer dan 7 |
|  | Toelichting | <open invulveld> |
| Had u last van braken? | | ja; nee |
|  | Hoe ernstig was het? | iets; matig; veel |
|  | Wanneer is dit begonnen? | <datum> |
|  | Hoeveel dagen heeft het geduurd? | 1; 2; 3; 4; 5; 6; 7; meer dan 7 |
|  | Toelichting | <open invulveld> |
| Had u last van harde buiken? | | ja; nee |
|  | Hoe ernstig was het? | iets; matig; veel |
|  | Wanneer is dit begonnen? | <datum> |
|  | Hoeveel dagen heeft het geduurd? | 1; 2; 3; 4; 5; 6; 7; meer dan 7 |
|  | Toelichting | <open invulveld> |
| Had u last van diarree? | | ja; nee |
|  | Hoe ernstig was het? | iets; matig; veel |
|  | Wanneer is dit begonnen? | <datum> |
|  | Hoeveel dagen heeft het geduurd? | 1; 2; 3; 4; 5; 6; 7; meer dan 7 |
|  | Toelichting | <open invulveld> |
| Had u last van duizeligheid? | | ja; nee |
|  | Hoe ernstig was het? | iets; matig; veel |
|  | Wanneer is dit begonnen? | <datum> |
|  | Hoeveel dagen heeft het geduurd? | 1; 2; 3; 4; 5; 6; 7; meer dan 7 |
|  | Toelichting | <open invulveld> |
| Had u last van een verminderde eetlust? | | ja; nee |
|  | Hoe ernstig was het? | iets; matig; veel |
|  | Wanneer is dit begonnen? | <datum> |
|  | Hoeveel dagen heeft het geduurd? | 1; 2; 3; 4; 5; 6; 7; meer dan 7 |
|  | Toelichting | <open invulveld> |
| Had u last van gewrichts- of spierstijfheid? | | ja; nee |
|  | Hoe ernstig was het? | iets; matig; veel |
|  | Wanneer is dit begonnen? | <datum> |
|  | Hoeveel dagen heeft het geduurd? | 1; 2; 3; 4; 5; 6; 7; meer dan 7 |
|  | Toelichting | <open invulveld> |
| Had u last van jeuk? | | ja; nee |
|  | Hoe ernstig was het? | iets; matig; veel |
|  | Wanneer is dit begonnen? | <datum> |
|  | Hoeveel dagen heeft het geduurd? | 1; 2; 3; 4; 5; 6; 7; meer dan 7 |
|  | Toelichting | <open invulveld> |
| Had u last van overmatig zweten? | | ja; nee |
|  | Hoe ernstig was het? | iets; matig; veel |
|  | Wanneer is dit begonnen? | <datum> |
|  | Hoeveel dagen heeft het geduurd? | 1; 2; 3; 4; 5; 6; 7; meer dan 7 |
|  | Toelichting | <open invulveld> |
| Had u last van huiduitslag? | | ja; nee |
|  | Hoe ernstig was het? | iets; matig; veel |
|  | Wanneer is dit begonnen? | <datum> |
|  | Hoeveel dagen heeft het geduurd? | 1; 2; 3; 4; 5; 6; 7; meer dan 7 |
|  | Toelichting | <open invulveld> |
| Had u last van gezwollen klieren in de nek, oksel of lies? | | ja; nee |
|  | Hoe ernstig was het? | iets; matig; veel |
|  | Wanneer is dit begonnen? | <datum> |
|  | Hoeveel dagen heeft het geduurd? | 1; 2; 3; 4; 5; 6; 7; meer dan 7 |
|  | Toelichting | <open invulveld> |
| Had u last van keelpijn? | | ja; nee |
|  | Hoe ernstig was het? | iets; matig; veel |
|  | Wanneer is dit begonnen? | <datum> |
|  | Hoeveel dagen heeft het geduurd? | 1; 2; 3; 4; 5; 6; 7; meer dan 7 |
|  | Toelichting | <open invulveld> |
| Had u last van verkoudheid? | | ja; nee |
|  | Hoe ernstig was het? | iets; matig; veel |
|  | Wanneer is dit begonnen? | <datum> |
|  | Hoeveel dagen heeft het geduurd? | 1; 2; 3; 4; 5; 6; 7; meer dan 7 |
|  | Toelichting | <open invulveld> |
| Had u last van hoesten? | | ja; nee |
|  | Hoe ernstig was het? | iets; matig; veel |
|  | Wanneer is dit begonnen? | <datum> |
|  | Hoeveel dagen heeft het geduurd? | 1; 2; 3; 4; 5; 6; 7; meer dan 7 |
|  | Toelichting | <open invulveld> |
| Had u last van flauwvallen? | | ja; nee |
|  | Hoe ernstig was het? | iets; matig; veel |
|  | Wanneer is dit begonnen? | <datum> |
|  | Hoeveel dagen heeft het geduurd? | 1; 2; 3; 4; 5; 6; 7; meer dan 7 |
|  | Toelichting | <open invulveld> |
| Had u last van griep of griepachtige klachten? | | ja; nee |
|  | Hoe ernstig was het? | iets; matig; veel |
|  | Wanneer is dit begonnen? | <datum> |
|  | Hoeveel dagen heeft het geduurd? | 1; 2; 3; 4; 5; 6; 7; meer dan 7 |
|  | Toelichting | <open invulveld> |
| Had u last van iets anders? Zo ja, vul in: | | <open invulveld> |
|  | Hoe ernstig was het? | iets; matig; veel |
|  | Wanneer is dit begonnen? | <datum> |
|  | Hoeveel dagen heeft het geduurd? | 1; 2; 3; 4; 5; 6; 7; meer dan 7 |
|  | Toelichting | <open invulveld> |
| **Vragen over de symptomen vóór de vaccinatie**  Als u op alle vragen over de symptomen 'nee' heeft ingevuld, dan kunt u de volgende vragen overslaan. | | |
| Heeft u medische hulp gezocht naar aanleiding van de symptomen? | | ja; nee |
|  | Hoe heeft u medische hulp gezocht? | telefoon huisarts; bezoek huisarts; bezoek ziekenhuis; opname in ziekenhuis; natuurgeneeskundige; extra contact met verloskundige of gynaecoloog; anders |
| Heeft u pijnstillers of medicijnen gebruikt in de week voor de vaccinatie? | | ja; nee |
|  | Welke pijnstillers? | <open invulveld> |
|  | Hoe lang heeft u de pijnstillers gebruikt in de week voor de vaccinatie? | 1 tot 2 dagen; 3 of meer dagen |
| Heeft u zich vanwege de symptomen ziek gemeld voor uw werk, in de week voor de vaccinatie? | | ja; nee |
|  | Hoe lang heeft u zich vanwege de klachten ziekgemeld van uw werk? | 1 tot 2 dagen; 3 of meer dagen |
| Vul in als er nog bijzonderheden waren in de week voor de vaccinatie: | | <open invulveld> |
| **Symptomen in de week ná de vaccinatie**  Geef hieronder aan of u symptomen had in de week ná de vaccinatie. Als u bij de symptomen 'ja' invult, beantwoord dan ook de vervolgvragen. | | |
| Had u last van koorts? | | ja; nee |
|  | Wat was de hoogst gemeten temperatuur? | 38 tot 39°C; 39 tot 40°C; 40 tot 41°C; hoger dan 41°C |
|  | Hoe is dit gemeten? | rectaal; oksel; oor; tast; anders; niet gemeten |
|  | Wanneer is dit begonnen? | <datum> |
|  | Hoeveel dagen heeft het geduurd? | 1; 2; 3; 4; 5; 6; 7; meer dan 7 |
|  | Toelichting | <open invulveld> |
| Had u last van hoofdpijn? | | ja; nee |
|  | Hoe ernstig was het? | iets; matig; veel |
|  | Wanneer is dit begonnen? | <datum> |
|  | Hoeveel dagen heeft het geduurd? | 1; 2; 3; 4; 5; 6; 7; meer dan 7 |
|  | Toelichting | <open invulveld> |
| Had u last van vermoeidheid? | | ja; nee |
|  | Hoe ernstig was het? | iets; matig; veel |
|  | Wanneer is dit begonnen? | <datum> |
|  | Hoeveel dagen heeft het geduurd? | 1; 2; 3; 4; 5; 6; 7; meer dan 7 |
|  | Toelichting | <open invulveld> |
| Had u last van misselijkheid? | | ja; nee |
|  | Hoe ernstig was het? | iets; matig; veel |
|  | Wanneer is dit begonnen? | <datum> |
|  | Hoeveel dagen heeft het geduurd? | 1; 2; 3; 4; 5; 6; 7; meer dan 7 |
|  | Toelichting | <open invulveld> |
| Had u last van braken? | | ja; nee |
|  | Hoe ernstig was het? | iets; matig; veel |
|  | Wanneer is dit begonnen? | <datum> |
|  | Hoeveel dagen heeft het geduurd? | 1; 2; 3; 4; 5; 6; 7; meer dan 7 |
|  | Toelichting | <open invulveld> |
| Had u last van harde buiken? | | ja; nee |
|  | Hoe ernstig was het? | iets; matig; veel |
|  | Wanneer is dit begonnen? | <datum> |
|  | Hoeveel dagen heeft het geduurd? | 1; 2; 3; 4; 5; 6; 7; meer dan 7 |
|  | Toelichting | <open invulveld> |
| Had u last van diarree? | | ja; nee |
|  | Hoe ernstig was het? | iets; matig; veel |
|  | Wanneer is dit begonnen? | <datum> |
|  | Hoeveel dagen heeft het geduurd? | 1; 2; 3; 4; 5; 6; 7; meer dan 7 |
|  | Toelichting | <open invulveld> |
| Had u last van duizeligheid? | | ja; nee |
|  | Hoe ernstig was het? | iets; matig; veel |
|  | Wanneer is dit begonnen? | <datum> |
|  | Hoeveel dagen heeft het geduurd? | 1; 2; 3; 4; 5; 6; 7; meer dan 7 |
|  | Toelichting | <open invulveld> |
| Had u last van een verminderde eetlust? | | ja; nee |
|  | Hoe ernstig was het? | iets; matig; veel |
|  | Wanneer is dit begonnen? | <datum> |
|  | Hoeveel dagen heeft het geduurd? | 1; 2; 3; 4; 5; 6; 7; meer dan 7 |
|  | Toelichting | <open invulveld> |
| Had u last van gewrichts- of spierstijfheid? | | ja; nee |
|  | Hoe ernstig was het? | iets; matig; veel |
|  | Wanneer is dit begonnen? | <datum> |
|  | Hoeveel dagen heeft het geduurd? | 1; 2; 3; 4; 5; 6; 7; meer dan 7 |
|  | Toelichting | <open invulveld> |
| Had u last van jeuk? | | ja; nee |
|  | Hoe ernstig was het? | iets; matig; veel |
|  | Wanneer is dit begonnen? | <datum> |
|  | Hoeveel dagen heeft het geduurd? | 1; 2; 3; 4; 5; 6; 7; meer dan 7 |
|  | Toelichting | <open invulveld> |
| Had u last van overmatig zweten? | | ja; nee |
|  | Hoe ernstig was het? | iets; matig; veel |
|  | Wanneer is dit begonnen? | <datum> |
|  | Hoeveel dagen heeft het geduurd? | 1; 2; 3; 4; 5; 6; 7; meer dan 7 |
|  | Toelichting | <open invulveld> |
| Had u last van huiduitslag? | | ja; nee |
|  | Hoe ernstig was het? | iets; matig; veel |
|  | Wanneer is dit begonnen? | <datum> |
|  | Hoeveel dagen heeft het geduurd? | 1; 2; 3; 4; 5; 6; 7; meer dan 7 |
|  | Toelichting | <open invulveld> |
| Had u last van gezwollen klieren in de nek, oksel of lies? | | ja; nee |
|  | Hoe ernstig was het? | iets; matig; veel |
|  | Wanneer is dit begonnen? | <datum> |
|  | Hoeveel dagen heeft het geduurd? | 1; 2; 3; 4; 5; 6; 7; meer dan 7 |
|  | Toelichting | <open invulveld> |
| Had u last van keelpijn? | | ja; nee |
|  | Hoe ernstig was het? | iets; matig; veel |
|  | Wanneer is dit begonnen? | <datum> |
|  | Hoeveel dagen heeft het geduurd? | 1; 2; 3; 4; 5; 6; 7; meer dan 7 |
|  | Toelichting | <open invulveld> |
| Had u last van verkoudheid? | | ja; nee |
|  | Hoe ernstig was het? | iets; matig; veel |
|  | Wanneer is dit begonnen? | <datum> |
|  | Hoeveel dagen heeft het geduurd? | 1; 2; 3; 4; 5; 6; 7; meer dan 7 |
|  | Toelichting | <open invulveld> |
| Had u last van hoesten? | | ja; nee |
|  | Hoe ernstig was het? | iets; matig; veel |
|  | Wanneer is dit begonnen? | <datum> |
|  | Hoeveel dagen heeft het geduurd? | 1; 2; 3; 4; 5; 6; 7; meer dan 7 |
|  | Toelichting | <open invulveld> |
| Had u last van flauwvallen? | | ja; nee |
|  | Hoe ernstig was het? | iets; matig; veel |
|  | Wanneer is dit begonnen? | <datum> |
|  | Hoeveel dagen heeft het geduurd? | 1; 2; 3; 4; 5; 6; 7; meer dan 7 |
|  | Toelichting | <open invulveld> |
| Had u last van griep of griepachtige klachten? | | ja; nee |
|  | Hoe ernstig was het? | iets; matig; veel |
|  | Wanneer is dit begonnen? | <datum> |
|  | Hoeveel dagen heeft het geduurd? | 1; 2; 3; 4; 5; 6; 7; meer dan 7 |
|  | Toelichting | <open invulveld> |
| Heeft u last gehad van pijn op de prikplek? | | ja; nee |
|  | Hoe ernstig was het? | iets; matig; veel |
|  | Wanneer is dit begonnen? | <datum> |
|  | Hoeveel dagen heeft het geduurd? | 1; 2; 3; 4; 5; 6; 7; meer dan 7 |
|  | Toelichting | <open invulveld> |
| Heeft u last gehad van roodheid op de prikplek? | | ja; nee |
|  | Hoe ernstig was het? | iets; matig; veel |
|  | Wanneer is dit begonnen? | <datum> |
|  | Hoeveel dagen heeft het geduurd? | 1; 2; 3; 4; 5; 6; 7; meer dan 7 |
|  | Toelichting | <open invulveld> |
| Heeft u last gehad van zwelling op de prikplek? | | ja; nee |
|  | Hoe ernstig was het? | iets; matig; veel |
|  | Wanneer is dit begonnen? | <datum> |
|  | Hoeveel dagen heeft het geduurd? | 1; 2; 3; 4; 5; 6; 7; meer dan 7 |
|  | Toelichting | <open invulveld> |
| Heeft u last gehad van een harde bult op de prikplek? | | ja; nee |
|  | Hoe ernstig was het? | iets; matig; veel |
|  | Wanneer is dit begonnen? | <datum> |
|  | Hoeveel dagen heeft het geduurd? | 1; 2; 3; 4; 5; 6; 7; meer dan 7 |
|  | Toelichting | <open invulveld> |
| Had u last van iets anders? Zo ja, vul in: | | <open invulveld> |
|  | Hoe ernstig was het? | iets; matig; veel |
|  | Wanneer is dit begonnen? | <datum> |
|  | Hoeveel dagen heeft het geduurd? | 1; 2; 3; 4; 5; 6; 7; meer dan 7 |
|  | Toelichting | <open invulveld> |
| **Vragen over de symptomen ná de vaccinatie**  Als u op alle vragen over de symptomen 'nee' heeft ingevuld, dan kunt u de volgende vragen overslaan. | | |
| Heeft u medische hulp gezocht naar aanleiding van de symptomen? | | ja; nee |
|  | Hoe heeft u medische hulp gezocht? | telefoon huisarts; bezoek huisarts; bezoek ziekenhuis; opname in ziekenhuis; natuurgeneeskundige; extra contact met verloskundige of gynaecoloog; anders |
| Heeft u pijnstillers of medicijnen gebruikt in de week na de vaccinatie? | | ja; nee |
|  | Welke pijnstillers? | <open invulveld> |
|  | Op welk moment heeft u de pijnstillers genomen? | 0-6 uur na de vaccinatie; 4-24 uur na de vaccinatie; 24-48 uur na de vaccinatie; langer dan 48 uur na de vaccinatie |
|  | Hoe lang heeft u de pijnstillers gebruikt in de week na de vaccinatie? | 1 tot 2 dagen; 3 of meer dagen |
| Heeft u zich vanwege de symptomen ziek gemeld voor uw werk, in de na voor de vaccinatie? | | ja; nee |
|  | Hoe lang heeft u zich vanwege de klachten ziekgemeld van uw werk? | 1 tot 2 dagen; 3 of meer dagen |
| Vul in als er nog bijzonderheden waren in de week na de vaccinatie: | | <open invulveld> |
